# Mapping the effectiveness of integrating mental health in HIV programs: a scoping review

**DOI:** 10.1186/s12913-023-09359-x

**Published:** 2023-04-24

**Authors:** Ndeshiteelela K Conteh, Ashley Latona, Ozayr Mahomed

**Affiliations:** 1 University of KwaZulu- Natal, Durban, South Africa; 2 Tufts School of Medicine, Boston, USA

**Keywords:** HIV, Mental Health, Mental health integration

## Abstract

**Introduction:**

Mental health and substance abuse issues are increasing among HIV-positive people, and it negatively impacts health outcomes like engagement, retention in HIV care, and adherence to ART. Thus, national ART programs must include mental health management. The scoping review sought to map evidence on the efficacy of combining HIV and mental health care.

**Methods:**

The Arksey and O’Malley methodological framework was used to map the existing research on integrating HIV and mental health services to identify knowledge gaps. Two independent reviewers screened articles for inclusion. Studies on HIV-mental health integration were considered. We searched numerous sources, extracted data, and summarized publications by integration model and patient outcomes.

**Results:**

Twenty-nine articles met the criteria for this scoping review. Twenty-three studies were from high-income countries, with only six from low and middle-income countries in Africa (Zimbabwe 1, Uganda 3, South Africa 1, and Tanzania 1). Most of the literature discussed single-facility integration although multi-facility and integrated care through a case manager was researched as well. There was a reduction in depression, alcohol use, increased social function, decreased self-reported stigma, decreased psychiatric symptoms, and improved mood in PLHIV who received cognitive behavioral therapy in settings implementing integrated care. When providing integrated mental health services to PLHIV, healthcare workers reported feeling more comfortable discussing mental illness. Personnel in the mental health field reported less stigma and increased PLHIV referrals for mental health services due to integrated HIV and mental health care.

**Conclusion:**

According to the research, integrating mental health services into HIV care improves the diagnosis and treatment of depression and other mental disorders related to substance abuse in PLHIV.

## Background

Human Immunodeficiency Virus and Acquired Immunodeficiency Syndrome (HIV/AIDS) are major public health issues globally, with high mortality rates in Sub-Saharan Africa. According to the Joint UN Programme on HIV/AIDS (UNAIDS), there were 38.4 million HIV-positive people worldwide in 2021, and about 1.5 million people acquired HIV[1]. In 2021, approximately 650,000 people died from HIV/AIDS compared to 2 million in 2004 [1], indicating significant progress in reducing HIV-related mortality.

Despite only having 17% of the world’s population, Sub-Saharan Africa accounts for over 70% of global HIV/AIDS cases[2]. HIV/AIDS incidence is 23% in South Africa, 15% in Nigeria, 10% in Uganda, 8% in Mozambique, and 7% in Kenya[2]. Namibia has one of the world’s highest HIV rates. The Namibian Demographic and Health Survey (DHS) 2013 indicated that the HIV prevalence among adults in Namibia was around 14%. In some Namibian regions, the HIV prevalence was as high as 22%. The Zambezi region has the highest HIV prevalence rate in Namibia, at 22.3% [3].

HIV-positive people’s mental health and substance abuse issues are rising [4] and a higher proportion of HIV-positive people have a mental illness[4]. Patients with mental health issues require a much longer continuum of care [4]. This is compared to 7% and 2% of the general population [4]. In Canada, 40% of PLWH had a mental illness, compared to 22% of HIV-negative people [5]. A multisite study in the United States of 2800 PLHIV found that 36% of patients had depression and 15.8% had a generalized anxiety disorder, respectively, compared to only 6.7% and 2.1% in the general population[4]. Furthermore, the study discovered a li between depression and adherence to antiretroviral therapy (ART); patients with depression had a 42% lower adherence[4].

Mental illnesses are common in PLHIV Africa. Mental illness affects between 26% and 38% of PLWH in South Africa, compared to 13% of the general population [6]. Mental health impairment can result in adverse health outcomes at any point along the HIV continuum of care[4]. Additionally, mental health disorders can obstruct adequate engagement and retention in HIV care[4], and depression is common in PLWHIV.

Significant improvements have been attained to increase antiretroviral treatment (ART) access and uptake in Namibia. With high ART coverage and retention in care in some countries, many PLHIV have improved their quality of life, enabling them to live healthy and productive lives. A growing body of evidence shows that HIV-positive people need mental health care, but it is often overlooked or untreated[7]. Psychological disorders such as depression, cognitive impairments, and personality disorders significantly impact adherence to ART and clinic attendance and quality of life[7]. If not addressed, these factors can speed up disease progression and spread [7]. With some countries almost attaining epidemic control, it is critical to be aware of the mental health needs of those on ART in the continuum of care and integrate mental health management as part of their regular programming.

Existing literature doesn’t seem to have one standardized and uniform definition of integration. However, most literature seems to align with the general WHO definition of defining integrated service delivery as “the management and delivery of health care services so that the clients receive a continuum of preventive and curative services that caters to their needs over time and across different levels of the health system”[8]. Furthermore, this definition does affiliate with the definition of integration as “managerial or operational changes to health systems to bring together inputs, delivery, management and organization of particular service functions as a means of improving coverage, access, quality, acceptability and (cost)-effectiveness[9].

The literature claims that there is an evident need for universal mental health screening and the provision of mental health therapy that is integrated into continuing HIV care, given the substantial evidence linking mental health and behavioral disorders to poor HIV health outcomes[4].

The review has outlined that, most people regardless of country setting do not receive the much-needed mental health care partly because they are not identified as having a mental health disorder[10–13]. Additionally, the findings highlight that the various factors contribute to this gap in mental health screening and treatment provision such as human resource shortages, fragmented forms of service delivery, and a lack of implementation and policy change capabilities. A significant obstacle that is noted is the stigma of mental illness that prevails at all levels, including among patients, healthcare professionals, and policymakers [4]. Communally, the literature around the integration of mental health services into HIV care indicates positive patient outcomes for PLHIV as data has shown improved clinical outcomes of both HIV and mental health disorders, reduction in substance use behaviors and stigma, improvements in social functioning, a higher patient engagement in care, reduced self-reported HIV stigma[4, 9].

With this substantial evidence, which is mostly from high-income country studies, one can only assume the greater need for mental health service integration in low-income countries such as Namibia.

This scoping review aimed to map evidence of the effectiveness of integrating HIV and mental health services.

### Objectives

The objectives of the scoping review are to:

1. Determine what models of integration exist.
2. Identify and map out models of integration and effects on patient outcomes.
3. Outline enablers and barriers to integration.

## Methods

A scoping review of the academic literature on integrating mental health services into HIV settings was conducted using the Arksey and O’Malley methodological framework[14]. The Arksey and O’Malley methodological framework aids in identifying relevant literature regardless of study type[14]. The scoping review framework consists of a stage process for conducting a comprehensive search of the evidence-based literature, and this framework was followed to map the integration of mental health services in HIV programs[14, 15].

### Identifying the research question

The review’s objectives were to assess existing knowledge and gaps regarding the effectiveness of mental health and HIV program integration and determine which model would be most appropriate for the Namibian context. Three research questions guided the review:

1. What models were used to manage mental illness in HIV-positive patients?
2. What were the patient outcomes with the assessed models of integration?
3. What are the facilitators and barriers to integration?

### Identifying relevant studies

Inclusion and exclusion criteria

#### Inclusion criteria

- Qualitative and quantitative studies describing or evaluating the integration of mental health and HIV and AIDS services.
- Studies on the integration of mental health services, including substance abuse and HIV among the adult population.

#### Exclusion criteria

- Articles that are not in English were excluded as the researcher couldn’t assess them due to the language barrier.

### Search strategy

The scoping review incorporated quantitative, qualitative, and mixed-methods research and systematic reviews and meta-analyses. The content eligibility criteria were developed per the JBI reviewer’s manual (2015)[16], which recommends using the mnemonic PCC (population, concept, and context) to narrow the review’s focus and scope (Table 1).

**Table 1:** **PCC Framework**

Criteria	Determinants
Population	adults who are on ART and have experienced any form of mental disorder, including substance abuse.
Concept	describe, implement, or provide guidance on a mental health intervention among PLHIV or evaluate patient outcomes due to using an integration model among PLHIV with mental illnesses.
Context	implementation and integration of mental health management models into HIV programs and settings.

For this scoping review, a literature search was conducted in MEDLINE, Academic search complete, APA PsycInfo, CAB, and Health Source/nursing academic databases which were accessed through the EBSCO search engine. The search was done using the search terms “HIV” AND “mental health,“ as well as “HIV and Mental health integration. This literature search was not restricted in any way by the type of study being conducted however the search was limited to articles in English and are published between 2004 and 2021.

The method used to conduct the literature search was adapted from previously published scoping review literature[17, 18], which described using two separate reviewers to conduct article screening. First, the reviewers did their search for article titles across a variety of databases by using the search terms, and then they made a list of the literature that satisfies the requirements by evaluating it based on the title and the abstract. Second, the two reviewers discussed which articles should be included in the scoping review and why. During the second part of the review, once the team had explored their differences and found ways to resolve them, they were able to establish a consensus. Third, the use of the Preferred Reporting Items for Systematic and Meta-Analyses ( PRISMA )2020 checklist[19] was applied to include articles that met the inclusion criteria and were deemed acceptable by both reviewers. We utilized the PRISMA checklist[19] to evaluate a combined list of publications that we had agreed upon based on our inclusion criteria and the differences between reviewers. The final list had duplicate articles eliminated, and a summary of the consensus was also recorded.

**Fig. 1:**
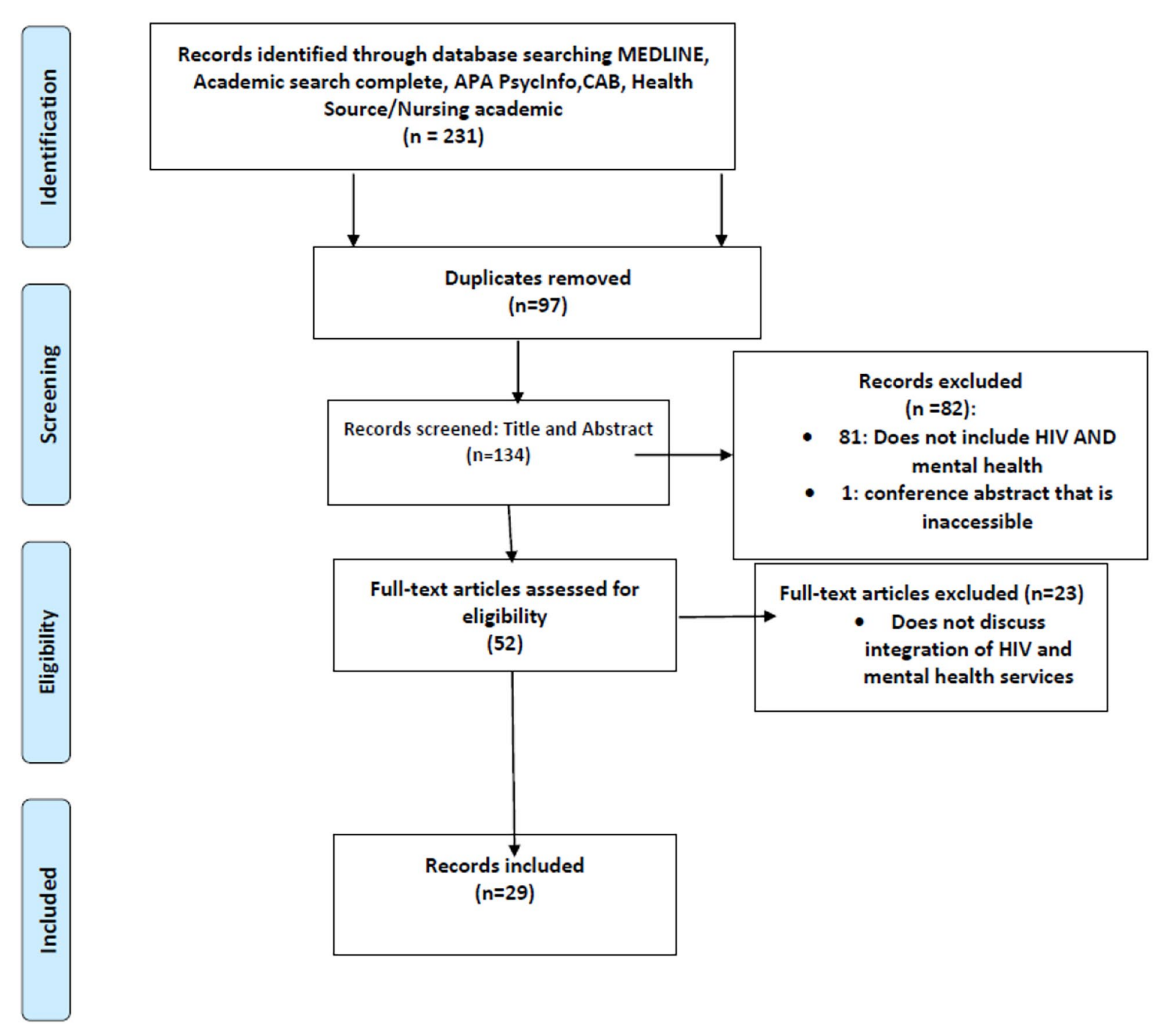
Study Selection diagram

The study selection diagram, Fig. 1, summarizes the literature search results, depicting the identification, and screening process used in the study selection process.

### Data items and data collection process

To facilitate comparison and thematic analysis, the following data were retrieved from the articles: integration model, authors and year of publication; study setting and sample size; study design; description of intervention; outcomes, and results. A table that provides a summary of the findings from the studies that were included in the analysis was developed (Table 2). The information that is currently known concerning the incorporation of mental health services into HIV treatment programs or settings for PLHIV is summarized in Table 2 below.

**Table 2:** **Descriptive characteristics of the final included studies**

Author, Year	Setting & Sample size	Study Design	Objective	Description of Intervention	Outcomes
**Model 1: Single facility Integration**					
Feldman et al., 2012	USA n = 314 clients of the AIDS organization	Retrospective record review (cohort)	To evaluate the Rapid Response System (a set of operating procedures designed to facilitate interdepartmental linkage of clients to mental health evaluation) in an AIDS service organization	Rapid Response System contact and evaluation appointment,	* 64% of clients completed health evaluation
Nakimuli-Mpungu et al., 2014	Uganda n = 500 PLHIVs	Cross-sectional	To examine the prevalence and cardinal demographic, psychosocial, and clinical features associated with having any depressive disorder, sub-clinical depression, current and lifetime depressive disorders among patients with human immunodeficiency virus (HIV) in southern Uganda.	N/A	*Revealed misperception about the etiology and treatment of depression * CBT technique deemed culturally acceptable
Dodds et al., 2004	USA	Service Article	N/A	N/A	N/A
Namata Mbogga Mukasa et al., 2014	Uganda 10,285 records of PLHIV	Longitudinal study	To assess the effectiveness of case finding and management of non-communicable diseases NCD	N/A	*Improved case finding of patients with NCDs
Esposito-Smythers et al., 2014	USA N = 17 young PLV	RCT	to test an integrated cognitive behavioral and (CBT/CM) intervention for young people living with HIV (YPLH) with an alcohol and/or cannabis disorder	Provision of Cognitive behavioral therapy	* Significant reductions in alcohol use, withdrawal symptoms, dependence symptoms, and related problems,
Surah et al., 2013	UK Ireland, UK. In-reach HIV clinic. n = 37 HIV-infected injecting drug users.	Intervention study (non-randomized	To evaluate integrated care versus standard care offered in a psychiatric led clinic	Provision of integrated care (HIV and Mental health0	*Clinical outcomes improved *Substance and alcohol misuse, HRQOL and Hospital Anxiety
Nebelkopf and Penagos et al., 2005	USA N = 45 PLHIV	Survey (pre/pos)	Assess the effectiveness of integrated mental health care	Provision of integrated HIV and mental health care	*Positive changes in quality of life
Wood 2008	USA	Case study	Discusses barriers to care for rural HIV-positive substance abusers, and challenges for rural health care providers	N/A	N/A
Tetrault et al., 2012	USA N = 47PLHIV	RCT	Assess the feasibility of Integrating buprenorphine/naloxone into HIV treatment settings	Provision of integrated naloxone treatment in HIV care	*Decrease in Viral load *No improvement in patient outcome detected
Coleman et al., 2012	USA n = 124 PLHIV	Retrospective cohort	To assess the effectiveness of an integrated, measurement-based approach to depression	Provision of psychopharmacologic and psychological therapies for PLHIV	*Reduced depression scores
Winiarski et a, 2005	USA n = 147 PLHIV	Non-randomized Intervention study	To evaluate the effectiveness of an HIV mental health program integrated into a primary health care setting. Emphasis is on cultural responsiveness	Provision of mental health services for PHIV	*Reduction in mental health problems *Reduced alcohol use *Improved social functioning
Farber et al., 2014	USA n = 48 PLHIV	Pre- and Post-intervention Cohort	To assess change in perceived stigma post-intervention	Provision of an integrated mental health program into community-based HIV primary care.	*Reduced self-reported perceived stigma
Vergara-Rodriguez et al., 2012	USA n = 123 dual diagnosis patients	Cohort study	To assess the effect of an integrated treatment program (H-STAR)	Provision of the H_STAR program	*Reduction in alcohol, heroin, and cocaine post-intervention
**Model 2: Multi-facility Integration**					
Rosenberg et al., 2010	USA N = 236 patients with mental illness, of which 19 are PLHIV	RCT	To assess the STIRR intervention designed to integrate infectious disease programming in mental health settings	Provision of infectious diseases, including HIV, in mental health clinic	*Likelihood of intervention group reducing substance abuse *Intervention group more likely to be tested for Hepatitis B & C *No reduction in risky behavior and no increase in HIV knowledge
Curran et al., 2011	USA N = 249 depressed PLHIV	Randomized Trial	To compare the depression collaborative care intervention to usual depression care.	Provision of collaborative care	-
Sternhell et al., 2012	Australia	Retrospective study	To describe the development and functioning of HIV and hepatitis C mental health in primary care service: a multidisciplinary team that works with local general practitioners (GPs)	N/A	N/A
Daughters et al., 2010	USA n = 3 case series	Case Series	To examine the integration of HIV and depression medication adherence program	Provision of cognitive-behavioral therapy for HIV-positive people who are substance users	*Improved depression, ART initiation, and adherence rates
Wood and Austin,2009	USA	Service article	To outline the treatment integration needs of HIV-positive substance abusers and describe how one empirically selected social service program originated and continues to assist a community-based approach to	N/A	N/A
Taylor 2005	USA	Report	To describe the development and progress of an HIV program that delivers care for HIV and Hepatitis C virus (HCV) positive injection drug users	Provision of integrated addiction, psychiatric, HIV, and HCV care	*Adherence to weekly visits was at 99% *no one stopped treatment because of ongoing drug use or addiction relapse
Duffy et al., 2017	Zimbabwe n = 30	Mixed Methods study	To assess the feasibility of implementing the Stepped-Care model between the community, traditional medicine practitioners, and Health facilities for PLHIV	Training of health care workers, traditional medicine practitioners’ essential information on mental health disorders including alcohol and substance use, the stepped-care mental health and HIV integrated approach, therapeutic communication, and referral procedures	*More PLHIV (> 80%) received a referral for mental health/psychological services *Increased comfort of healthcare workers to discuss mental illness with patients *Reduced stigma among healthcare workers
**Model 3: Integration through care-coordination**					
Andersen et al., 2012	South Africa n = 14 HIV positive	Qualitative	To evaluate a nurse-delivered cognitive-behavioral therapy among PLHIV	Provide cognitive behavioral therapy for adherence to depression among ART users	*Reduction in depressive symptoms and level of impairment
Sacks et al., 2011	USA n = 76	RCT	To evaluate an integrated therapeutic community aftercare program in which clients learned to coordinate service components (HIV, mental health, and substance abuse) and integrate their treatment.	Provision of a modified therapeutic community aftercare program for PLHIV and are diagnosed with substance and mental disorder	*Moderate treatment effects with substance abuse
Bouis et al., 2007	USA n = 141 Triply diagnosed patients (HIV, mental illness, and substance (4)abuse)	Mixed-Method Study	To assess the effectiveness of an intervention that addresses the behavioral health care needs of HIV-infected individuals with both mental health and substance use problems	Provision of mental health and substance abuse management services in PLHIV	*Decrease in substance abuse *Reduction of psychiatric symptoms Increase inappropriate medicine use
Adams et al. 2012b	USA N = 3 academic clinic	Randomized Control Trial	To design an evidence-based approach to integrate depression care into HIV care	The use of measurement-based care (MBC) to track and assess antidepression tolerance using nonphysician depression care managers	*Facilitated provision of quality antidepressant management
Zaller et al., 2007	USA 116 PLHIV	Evaluation study	To assess a model of integrated substance-use counseling and referral for treatment within a primary care HIV-care	The use of a model of integrated substance abuse counseling and referral	* Success in assessing the substance use and mental health needs of HIV-infected individuals with numerous co-morbidities *Success in referrals
Sullivan et al., 2015	USA N = 21 PLHIV	Qualitative study	To explore patients’ experiences working with the nurse guide	N/A	* Properly trained nurse in this role can provide critical medical and psychosocial support to eliminate barriers to engagement in HIV care, and successfully facilitate patient HIV self-management
Odokonyero et al., 2015	Uganda N = HIV 10 clinics	Evaluation/Survey	To evaluate a task-sharing, protocolized approach to providing antidepressant care in HIV clinics in Uganda.	N/A	* Benefits of task shifting in LMIC
Adams et al. 2012a	Tanzania N = 20 HIV patients Tanzania. Outpatient HIV care and treatment center	Cohort	To test the feasibility of a task-shifting model of measurement based depression care in an HIV clinic	N/A	*Decreased depression score *Improved physical, social and mental health function
Adams et al. 2011	USA n = 144	Survey	To assess the feasibility of integrated depression care	Screen PLHIV for depress	* Reasonable feasibility in terms of identifying persons with depression * 31 patients (45%) screened positive for depression

### Consultation with stakeholders

This scoping review did not involve any stakeholder engagement. Additionally, because this was a scoping review, it was inappropriate to include patients or members of the public in the study.

## Results

This scoping review included 29 articles that met the criteria. Six of the 29 studies included in the scoping review were from Sub-Saharan Africa (Zimbabwe 1, Uganda 3, South Africa 1, and Tanzania 1). while 23 were from high-income countries (USA21, UK 1, Australia 1).

The literature suggests that integrating mental health services, particularly cognitive behavioral therapy, benefits PLHIV. The review found that including cognitive behavioral therapy in PLHIV with mental health issues reduced depression[20, 21], decreased alcohol use [22–24], increased social function[25], decreased self-reported stigma[26], reduced psychiatric symptoms, and improved mood[27]. The literature also shows that integrating mental health services into HIV care benefits ART providers. Healthcare workers providing integrated mental health services to PLHIV reported that they felt more comfortable discussing mental illness with patients[28]. Also, healthcare workers reported less stigmatization of mental illness and integrated mental health and HIV care also increased PLHIV referrals for mental health services[28].

### Models of integration

**Fig. 2:**
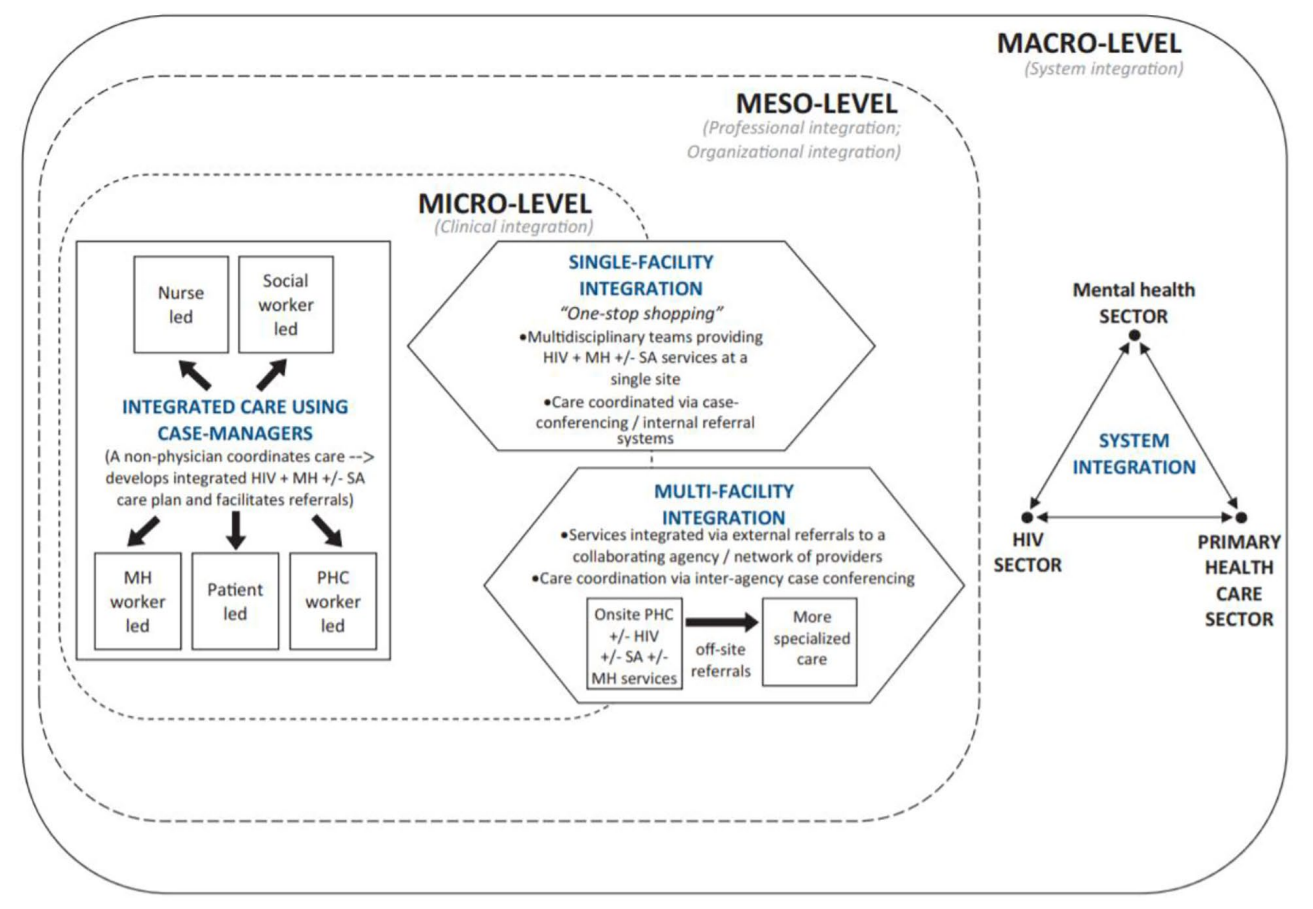
Integration models for HIV, mental health, and substance abuse services[9]

This review discovered no standard definition of integration in literature and only a few papers defined it. When defined, collaborative or coordinated care was used interchangeably to denote similar service delivery approaches. From co-location of services to coordinated care along a continuum including referrals and links via inter-agency collaborations, definitions vary. This suggests either the need for a standardized definition or on the contrary suggests that integration can be defined contextually, and no context is the same. However, the literature reviewed has outlined 3 integration models (Fig. 2): single-facility integration, multi-facility integration, and system integration. These models have been utilized to integrate mental health care into HIV services primarily to (1) increase mental health screening and treatment in ART clinics, (2) incorporate HIV care into mental health clinics, and (3) develop specific sub-specialty clinics serving persons with HIV and mental illness[9]. Three models that have integrated HIV and mental health at micro and Meso levels have been reviewed:

#### Model 1: single facility integration

The single facility integration model is also known as a “one-stop-shop” model where patients access various services as a single site. The single-facility integration model brings together a multidisciplinary team to offer comprehensive services to a patient. Various studies have implemented and evaluated the benefits of a single facility integration in addition to exploring the setup. In a study conducted in the US, a network of services was established to offer a comprehensive, holistic, and culturally competent system of care to people with substance abuse issues, mental illness, and HIV[29]. The network uses an approach that has four levels of care that are conceived in a pyramid structure. The lowest level of care is community outreach, followed by case management, outpatient counseling, and residential treatment[29]. In another setting in the US, mental health care services were integrated into the medical clinic where HIV care is provided. Just like the earlier described study, cultural appropriateness was highlighted as an important factor for consideration. Cultural appropriateness in this regard is considered to be mental health services acknowledging patients’ cultural identities and taking their beliefs, norms, and values into account when providing mental health intervention[30]. Single-facility integration also involved healthcare workers having individual discussions, using voicemails, and sharing medical notes to manage patients[30]. Another single-facility integration model described an internal referral system where care is managed between departments in the same facility. Coordination between departments can be enhanced by codified protocols that outline how coordination will take place[31].

Single-facility models are perceived to improve communication between providers and reduce scheduling and coordination time by health service providers[20]. This integration model has been associated with reduced access barriers, especially transportation, which is the main reason for limiting continuous access to care[20]. This integration model also improves confidentiality, which may be breached when someone is seen receiving care at a mental health or HIV facility, reducing stigma and easing some of the anxiety that people seeking care may experience[20, 32].

There have also been some disadvantages associated with this model of care. Literature has suggested that it may be more challenging to implement single-facility integration in rural areas or small cities where resources are limited[9] Providing a full continuum, of care within one health facility may not be cost-effective and practical, especially for patients with multiple co-morbidities needing comprehensive and specialized health care services[33].

#### Model 2: multi-facility integration

Multi-facility integration involves the integration of services at micro as well as meso- levels. Integration (professionally and organizationally) is achieved by collaborating with different agencies via collaborative networks and referral mechanisms. Clinical integration occurs through interagency case conferences as well as joint consultations. With Multi-facility integration, a facility may offer a range of services co-located at one site, and more specialized services are coordinated with other agencies[28, 34, 35]. The multi-facility integration can also be where services are integrated via interagency collaborations or mechanisms for external referrals to an intermediary. An intermediary can be a collaborating agency or even a network of offsite providers providing specialized mental health or HIV services[35–38].

In terms of providing comprehensive services to patients with complex needs, multi-facility integration is associated with practicality and cost-effectiveness from the provider’s perspective[9]. For patients with complex medical and social needs who require more comprehensive care, it is not feasible to provide an entire continuum of care at one site[39]. It is therefore more practical for patients who require comprehensive care to form collaborative network agencies One of the perceived disadvantages of multi-facility integration is that patients requiring care from multiple medical providers may receive inconsistent, coordinated care[35].

#### Model 3: integration through care coordination using case managers

This type of integration uses a non-physician. Case managers are nurses or social workers who develop treatment plans and facilitate referrals. In some studies, the nurse led the coordination of care[21, 40–42] and in others, care was coordinated by primary care staff[43–45] or a social worker[27, 46, 47]. Patients were also educated on how to navigate services and given tools to manage and monitor critical elements of their treatment progress and support was provided to patients to navigate self-help strategies and support groups. This kind of support empowers patients and enables them to adhere to key elements of their treatment[48]. Additionally, client involvement in treatment plans is seen as helpful in empowering patients and bridging care coordination gaps.

Integration through the care coordination model is reported to help patients access resources, psychosocial support, and education on how patients interact with doctors as well as create an opportunity for patients to seek clarification about health care information received[40]. Another perceived advantage of using case managers to coordinate care is its ability to promote a continuum of care as patients have a relationship with their case manager; although maximally achieving this advantage requires the case managers to initiate collaborations[9]. Intriguingly, this model of integration is reported to potentially address the challenge of underdiagnosis of depression in PLHIV, address antidepressant-antiretroviral interactions, and facilitate quality antidepressant management in HIV care as coordinators are always supervised by a psychiatrist[25].

The disadvantage of this model is that case managers’ appropriate professional training is essential to the ability of the case manager to initiate collaborations. The ability of the case manager to initiate collaboration can be hindered by other competing priorities of other service providers from different disciplines[27].

### Integrated HIV and mental health care: effects on patient outcomes

#### Model 1: single facility integration

Among the seven studies, some analyzed particular techniques like the measurement-based approach to depression care[20] while others evaluated operational systems to allow inter-organizational referrals[31]. Four studies examined outcomes before and after intervention ([20, 23, 26, 49], and one retrospectively assessed clinic data of a patient cohort upon referral completion[31]. Collectively, these studies [20, 26, 30, 31, 49] reported improvements in clinical outcomes of HIV and mental health disorders, reduction in substance use behaviors and stigma, improvements in social functioning, and higher patient engagement in care, although the overall risks of bias of the studies were high or unclear[9].

Two further investigations of integration within a single location were nonrandomized intervention trials [22, 30]. In a study that was carried out in the United States, 47 people living with HIV who were a part of the treatment group were compared to 100 people living with HIV who were a part of the control group[30]. The treatment group received integrated mental health, HIV, and primary care services that were designed to be culturally responsive and were co-located within a single site. The control group had access only to usual care, which included mental health services that were not HIV-specific and were not co-located with primary care. Higher use rates were seen among those receiving therapy, which was related to a lower incidence of mental health issues[30].

The other intervention trial was a non-randomized one, and it compared integrated care to standard care for HIV-infected IV drug users who were seeking treatment in an HIV clinic in Ireland that was overseen by psychiatrists who specialized in addiction treatment. Clients were recruited to participate in the study at thirty for the intervention group and twenty-six for the control group.

Although there were no significant changes in health-related quality of life (HRQOL), anxiety, depression, or drug abuse across the groups, the intervention group had a substantial improvement in clinical results[22].

#### Model 2: multi-facility integration

In yet another study, researchers used a combination of research approaches to investigate whether or not it would be possible to implement a Stepped-Care Model that would integrate the provision of services among communities, traditional medicine practitioners, and health facilities by employing standard operating procedures and trainer manuals. The survey that was conducted for this study demonstrated a high proportion of effective referrals (80–100%), as well as enhanced knowledge and reduced stigma among healthcare workers in treating patients who had co-morbidities[28].

The findings of three studies that evaluated programs that involved the integration of several facilities produced outcomes that reflected one or more indicators of efficacy. One study assessed the integration of a combined depression and HIV medication adherence program comprising three case series, and it found improvements in depression rates, the start of HAART, and medication adherence[35].

The third research was a randomized controlled trial that looked at the efficacy of the STIRR intervention, which stands for “Screening and Testing for HIV, Immunization against Hepatitis A and Risk Reduction.“

The third study is a randomized controlled trial (RCT) that evaluated the STIRR intervention. This intervention included HIV screening and testing, immunization against hepatitis A and B, risk reduction counseling, and referral and support for medical treatment. This intervention’s goals were to promote client acceptability of integrated infectious disease programs in mental health settings and to make such programs easier to implement in those settings[34]. The study enrolled a total of 236 clients with dual diagnoses who were undergoing treatment at a community mental health center. Participants were given a random assignment to either the STIRR intervention group (n = 118) or the control group (n = 118). The group serving as the control was given an improved version of the standard therapy. This includes immunization against hepatitis A and B, information on blood-borne infections, information on local community health sources for blood testing, and treatment as necessary. Subjects who were assigned to STIRR had high levels (over 80%) of participation and acceptance of core services, and they were more likely to be tested for hepatitis B and C (88% vs. 14% at 6 months); immunized for hepatitis A and B (76% vs. 5% at 6 months); have an increase in their hepatitis knowledge and reduce their substance abuse[34].

#### Model 3: Integration through care coordination using case managers

Two randomized controlled trials were connected to this integration model. One of the goals was to analyze an integrated therapeutic community aftercare program in Philadelphia, United States, for people with three separate diagnoses. The intervention group, which got integrated care, had 42 individuals allocated to it (55%), whereas the control group, which received conventional aftercare services, had 34 subjects assigned to it (45%). The intervention consisted of health and self-management groups, peer-support groups, self-help groups, individual case assistance, and family support groups. The goals of the intervention were to ensure treatment continuity and to assist patients in their transition to more independent functioning in the community. Those individuals in the study who started in better mental and physical health than the other participants experienced a larger overall improvement in their mental health and decreased their drug use if they were assigned to the intervention group rather than the control group[48].

Five studies that evaluated programs that included integration by a case manager reported findings reflecting one or more measures of effectiveness, with three of those studies focusing on feasibility. The first study used a qualitative approach to evaluate a cognitive behavioral therapy-based intervention in an integrated program. It found significant improvements in participants’ levels of depressive symptoms, global distress, and overall impairment[50]. The other trial was a cohort study that aimed to examine the feasibility of a task-shifting paradigm of measurement-based depression management. The researchers reported a reduction in depression score measured with PHQ-9 from 19.76 at baseline to 8.12 at week-12[25].

A non-randomized intervention trial that investigated the feasibility of a collaborative depression treatment model that uses social workers to coordinate care revealed that there was a decrease in depression ratings recorded during the course of the study[47]. People whose depression was more severe benefitted the most from case management, which had a significant impact not only on their physical, social, and mental well-being but also on the risk behaviors they engaged in. In addition, an association was found between the participants’ utilization of community services and a reduction in total expenditures for direct health and social services[46].

### Enablers and barriers to HIV integration

The literature in the scoping review has found overarching thematic areas or factors that play a role in the integration of HIV and mental health services. These factors can either be an enabler or a hindrance to the integration of HIV and mental health care as they can impact how health care systems operate.

- Collaborations and relationships.

Findings highlighted the importance of collaborations and relationships (both formal and informal) among healthcare providers, families, and communities. Particularly, families and communities were highlighted to be very important when integrating with services for mental health issues and substance use disorders in raising awareness and peer education [29, 51]. Good communication was highlighted as important in fostering collaboration and relationships, although ways of achieving good communication vary according to context, institutional processes, culture, and norms[33]. Co-location, although not sufficient on its own can facilitate communication but may hamper communication collaboration[52]. Effective information sharing, using electronic record systems, or having data/information sharing agreements facilitated communication, while restrictive rules and regulations to information sharing may be a barrier [53].

- Health workers, availability, roles, and incentives.

The availability and placement of healthcare workers that are trained appropriately is a facilitator, likewise, the lack of trained staff is a barrier to successful integration. Literature from LMIC countries highlights the lack of trained staff, staff shortages, and even high staff turnover as a barrier[54]. Task sifting, training health care workers by specialists, and support supervision can facilitate integration[54]. In some contexts, financial incentives were provided to healthcare providers to help with staff retention challenges[55] or participate in an integrated program[56].

- Institutional structures and resources to support integration.

Literature highlights that the right physical structures, commodities, and funding are facilitators of integration, likewise, a lack of such can also be a barrier to integration. Location and co-location for integration are very important facilitators and therefore accessibility and appropriateness of the location should be assured[54]. Evidence from studies highlights the benefit of integration to patients and clients, not only from the convenience and saving time by only having to attend one appointment, but also the collateral benefits of opportunistic screening and identification of diseases[57]. Additionally, another study[32] noted the benefits in terms of scheduling and reducing transport time as well. Data from the study cautions that co-location can be a barrier if not implemented sensitively and appropriately as it could affect confidentiality[53, 58, 59].

- Leadership, stewardship, management, and organizational culture.

Data reports that leadership including the political will to implement integration is a facilitator[60]. Articulating integration as a well-defined objective, conceiving of it as a desirable future reality, and coming up with a plan to bring this vision to life are all essential steps that demonstrated political will and this is exemplified by the fact that high-level policymakers have shown their dedication and support for the goal [60, 61]. Integration is made easier when structural and program design aspects permit these strategies. This includes senior management support for integrated models at the operational level in leading facilities[36, 56]. Strong leadership can ensure that this vision is shared by a diverse range of stakeholders[62, 63] while also recognizing and rewarding effective locally-led efforts[54]. This is especially important during scale-up when the viability and sustainability of specific models positively influence their diffusion[64, 65] and encourages lesson learning [60]; buy-in from frontline managers and staff is regarded as a critical facilitator.

## Discussion

The scoping review gives and snapshots overview of the available literature on the integration of HIV and mental health services. The findings of the literature highlight three main models used in HIV and mental health integration:1) facility integration, 2) multi-facility integration, and 3) integration through care coordination using case managers. The review also highlighted the advantages and disadvantages of each model regarding patient-related benefits and healthcare worker benefits.

Although the data presented in the literature is not sufficient to draw firm conclusions, health facility integration is reported to benefit patients more as services are offered under one roof, especially in sparsely populated settings where patients travel long distances to access services. Single facility model integration has the potential to reduce additional costs, especially transport costs to the patient, inconvenience [20, 32, 33], and improve physical access to health care services. The literature findings in this review that were derived from settings implementing single facility integration have highlighted the importance of having an integrated system of care that is culturally competent to the beneficiaries for it to be impactful to patients[30].

On the other hand, the literature suggests that patients with multiple co-morbidities may benefit from multi-facility integration as it is more effective and less costly, especially if the patient requires specialized care and mental health specialists are few [33]. Multi-facility integration requires effective collaboration and referrals to support positive patient outcomes, therefore health systems with fragmented referral mechanisms may not yield positive outcomes with this type of model.

Literature from this review suggests that the third model of integrated care coordinated by a case manager might be befitting in LMIC settings, however, healthcare providers need to be adequately trained in HIV and mental health management. Specifically, the findings highlight that less specialized cadres such as nurses and medical assistants can be trained to detect, screen, and manage psychological conditions under the supervision of a psychiatrist[25, 42]. In LMIC specifically, task shifting will be needed to effectively implement the integrated care coordinated by a case manager, especially since mental health specialists are limited.

Finally, the literature in this review clearly outlines some enablers and barriers related to integration. The presence of these factors can facilitate successful integration while on the other side, the absence of therefore creates barriers to integration. Effective collaborations including interdepartmental, and institutions are key to implementing all models of integration. The literature highlights that the rise and fall of any type of integration lie with the leadership and culture in health care systems[60] and it will require buy-in from policymakers [61].

In addition, the research that has been done has shown that resources, or even a lack of them, may either encourage or discourage integration. As a result, governments need to commit resources and invest to have effective integrated services that will improve patient outcomes. To do this, the government would have to make certain that integration is a strategy that is represented in national objectives and that there is buy-in from stakeholders that are pertinent to the support of integration. These resources are necessary for paying for the development of physical structures that will facilitate integration, as well as funding the technology that will be required, staff training, and/or recruiting, with the capacity of personnel being the most important of these.

### Study Limitations and Gaps

The bulk of research identified by the search originated from high-income nations, primarily the United States, which may be ascribed to publication bias. Due to the lack of published literature on LMICs, it is inappropriate to generalize conclusions. Also, a rigorous approach was not applied to retrieve literature, therefore literature included in this scoping review may not be extensive. There is a need for additional research to understand and quantify the investment/costs associated with the various existing integration strategies.

## Conclusion

Evidence from low- and middle-income countries (LMICs) is limited. However, the available evidence suggests that the integration of mental health services in HIV care settings does have a positive impact on PLHIV. The study findings show that integration of mental health services improves the diagnosis and management of mental illnesses such as depression and other mental disorders resulting from substance abuse in PLHIV. Study findings show that the integration of cognitive behavioral therapy at health facilities serving PLHIV has the greatest potential to reduce depressive symptoms and improve the overall psychosocial well-being of patients. Importantly, regardless of which model of care is used for integration, it is critical to consider the local context. It is vital to consider available resources (both financial and human), the continuum of care (diagnosis, treatment initiation, care for other morbidities), culture, institutional and social norms, and the continuum of care (diagnosis, treatment initiation, care for other morbidities) and most importantly, political will and leadership that supports integration.

## Data Availability

All data generated or analyzed during this study are included in this published article.
